# International survey on the implementation of the European and American guidelines on disorders of consciousness

**DOI:** 10.1007/s00415-023-11956-z

**Published:** 2023-09-23

**Authors:** Michele Farisco, Rita Formisano, Olivia Gosseries, Yoko Kato, Shigeki Koboyashi, Steven Laureys, Nicolas Lejeune, Charlotte Martial, Amal Matar, Ann-Marie Morrisey, Caroline Schnakers, Maidinamu Yakufujiang, Tomohiro Yamaki, Vigneswaran Veeramuthu, Matteo Zandalasini, Nathan Zasler, Alfonso Magliacano, Anna Estraneo, IBIA Special Interest Group on DoCs

**Affiliations:** 1 Centre for Research Ethics and Bioethics, Uppsala University, Uppsala, Sweden; 2 Biogem, Biology and Molecular Genetics Research Institute, Ariano Irpino, AV Italy; 3 IRCCS Santa Lucia Foundation, Rome, Italy; 4 Coma Science Group, GIGA-Consciousness, University of Liège, Liège, Belgium; 5 Centre du Cerveau, University Hospital of Liège, Liège, Belgium; 6 Department of Neurosurgery, Fujita Health University Bantane Hospital, Nagoya, Aichi Japan; 7 Division of Neurosurgery, Rehabilitation Center for Traumatic Apallics Chiba, National Agency for Automotive Safety and Victims’ Aid, 3-30-1 Isobe, Mihamaku, Chibashi, Chiba 261-0012 Japan; 8 CERVO Brain Research Center, University of Laval, Québec, QC Canada; 9 CHN William Lennox, Ottignies-Louvain-La Neuve, Belgium; 10 Institute of NeuroScienceUCLouvain, Ottignies-Louvain-La Neuve, Belgium; 11 School of Allied Health, Faculty of Education and Health Sciences, Ageing Research Centre, Health Research Institute, University of Limerick, Limerick, Ireland; 12 Research Institute, Casa Colina Hospital and Centers for Healthcare, Pomona, CA USA; 13 Thomson Hospital Kota Damansara, Petaling Jaya, Selangor Malaysia; 14 Unità Spinale, Neuroriabilitazione E Medicina Riabilitativa Intensiva, Dipartimento Di Medicina Riabilitativa, Azienda USL Di Piacenza, Piacenza, Italy; 15 Concussion Care Centre of Virginia, LTD, Henrico, VA 23233 USA; 16 Department of Physical Medicine and Rehabilitation, Virginia Commonwealth University, Richmond, VA 23298 USA; 17 IRCCS Fondazione Don Carlo Gnocchi ONLUS, Florence and Sant’Angelo dei Lombardi, AV, Italy

**Keywords:** Coma, Disorders of consciousness, Vegetative states, Unresponsive wakefulness syndrome, Minimally conscious state, Cognitive–motor dissociation, Neuroethics, Bioethics, Ethics, Clinical guidelines

## Abstract

Diagnostic, prognostic, and therapeutic procedures for patients with prolonged disorders of consciousness (pDoCs) vary significantly across countries and clinical settings, likely due to organizational factors (e.g., research vs. non-academic hospitals), expertise and availability of resources (e.g., financial and human). Two international guidelines, one from the European Academy of Neurology (EAN) and one from the American Academy of Neurology (AAN) in collaboration with the American Congress of Rehabilitation Medicine (ACRM) and the National Institute on Disability, Independent Living, and Rehabilitation Research (NIDILRR), were developed to facilitate consistent practice among professionals working with this challenging patient population. While the recommendations of both guidelines agree in principle, it remains an open issue how to implement them into clinical practice in the care pathway for patients with pDoCs. We conducted an online survey to explore health professional clinical practices related to the management of patients with pDoCs, and compare said practices with selected recommendations from both the guidelines. The survey revealed that while some recommendations are being followed, others are not and/or may require more honing/specificity to enhance their clinical utility. Particular attention should be given to the implementation of a multimodal assessment of residual consciousness, to the detection and treatment of pain, and to the impact of restrictions imposed by COVID-19 pandemics on the involvement of patients’ families/representatives.

**Supplementary Information:**

The online version contains supplementary material available at 10.1007/s00415-023-11956-z.

## Introduction

Research on patients with severe acquired brain injury and prolonged disorders of consciousness (pDoCs) has exploded in the last decade. However, the care approaches vary widely across countries and consequently may result in differences in patient management and outcomes [1, 2]. Standardized recommendations for management of individuals with pDoCs care should facilitate not only more consistent but also more effective clinician practice [3]. So far, the recent guidelines by the European Academy of Neurology (EAN) [4] (i.e., EU guidelines) and by the American Academy of Neurology (AAN) in collaboration with the American Congress of Rehabilitation Medicine (ACRM) and the National Institute on Disability, Independent Living, and Rehabilitation Research (NIDILRR) [5, 6] (i.e., US guidelines) are the most ambitious international attempts in this direction. In fact, they provide a wide-ranging list of recommendations for diagnosis, prognosis, and treatments of individuals with pDoCs based on state-of-the-art evidence from empirical research.

The US guidelines are an update of the 1994 American Task Force statement on persistent/permanent vegetative state (PVS) [7] and the 2002 clinical criteria of the minimally conscious state (MCS) [8]. The US guidelines provided more extensive recommendations for overall management of patients with pDoCs [5, 6], by stressing the importance of a multimodal assessment and multidisciplinary care in specialized neurorehabilitation settings to increase the chances of recovery. They also included a focus on patient and family needs, and caregivers and family counseling about patient diagnosis, prognosis, and treatment, and highlighted the importance of detecting pain and suffering in patients with pDoCs, as well as erring on the side of treatment even in situations where conscious awareness of pain was not clearly evident. Importantly, they proposed to replace the old term “permanent VS” as strictly related to the irreversibility of this clinical condition, with “chronic VS/UWS” (vegetative state has been renamed “unresponsive wakefulness syndrome” (UWS) by [9]) along with the time since brain injury.

In parallel, the EU guidelines aimed to offer recommendations for improving the diagnosis of pDoCs, with specific reference to data from a multimodal and multidisciplinary assessment by combining bedside clinical examination, neuroimaging, and neurophysiological evaluations (i.e., EEG) [4]. Additionally, the EU guidelines referred to advanced quantitative analysis of sleep, cortical responses to passive/resting-state paradigms measured by functional neuroimaging or neurophysiology, and complexity measures as the Perturbational Complexity Index (PCI) as promising strategies for detecting residual, not clinically evident, conscious activity in individuals with covert awareness [10, 11].

However, practical implementation of these guidelines might encounter several issues, such as limited financial and human resources in ordinary clinical settings, or limited access to the most up-to-date technology [12, 13], with both clinical and ethical relevance [14]. In this context, the Special Interest Group on Disorders of Consciousness of the International Brain Injury Association (IBIA-DoC-SIG;

https://www.internationalbrain.org/membership/ibia-special-interest-groups/disorders-of-consciousness-special-interest-group) launched an international online survey to investigate the practice trends of professionals working with patients with pDoCs in relation to selected recommendations from the two above-mentioned guidelines.

## Methods

### Survey questionnaire

A questionnaire (in English) was collaboratively designed in 2021 by an international task force including 18 IBIA-DoC-SIG members with expertise in DoCs and from neurology, neurosurgery, physical medicine and rehabilitation, neuropsychology, neuroscience, and ethics backgrounds.

The questionnaire targeted practices and opinions of professionals working with patients with pDoCs about selected recommendations from the EU and the US guidelines. Additional questions about the impact of COVID-19 pandemic on care for patients with pDoCs were added. The draft of the questionnaire was shared within the IBIA-DoC-SIG Task Force over a period of 1 year and discussed during several virtual meetings.

Table 1 reports the relevant recommendations from EU and US guidelines, and the respective questions asked in the present survey.

**Table 1:** Guidelines recommendations and related questions asked in the survey

EU guidelines recommendations	US guidelines recommendations	Questions asked
**Diagnosis**		
They indicate three sources of data for diagnosing DoCs: bedside examination (i.e., behavioral assessment at the bedside through clinically validated scales), functional neuroimaging, and EEG		What DoC diagnostic tools do you use in your practice?
“Repeat clinical assessments in the subacute and chronic setting, using the Coma Recovery Scale – Revised” “The classification of consciousness levels should never be made based on an isolated assessment”	“Perform serial standardized assessments to improve diagnostic accuracy” “To reduce diagnostic error in individuals with prolonged DoC after brain injury, serial standardized neurobehavioral assessments should be performed with the interval of reassessment determined by individual clinical circumstances”	- How often is the clinical assessment repeated in your diagnostic protocol? - Do you practice regular patient follow-ups? If yes, how often? - What are the factors impacting the frequency of the follow-ups? - Has COVID-19 pandemics changed your practice in this regard?
	“Clinicians should attempt to increase arousal before performing evaluations to assess level of consciousness anytime diminished arousal is observed or suspected”	-Do you usually try to prime the patients’ arousal level before assessing their level of consciousness? If yes, how?
“Despite the absence of eligible studies, spontaneous motor behavior and automatic motor responses may be observed and documented in the patient charts”		- Do you observe the patient prior to actually doing your hands-on clinical assessment?
“Whenever feasible, consider positron emission tomography, resting-state functional magnetic resonance imaging (fMRI), active fMRI or EEG paradigms and quantitative analysis of high-density EEG to complement behavioral assessment in patients without command following at the bedside” “The CRS-R be used to classify the level of consciousness”		- Which of the following clinical tools do you usually use? (may choose more than one): Coma Recovery Scale-Revised (CRS-R) Full Outline of Unresponsiveness Score (FOUR) Nociception Coma Scale-Revised (NCS-R) Other (please specify) - Which of the following technological assessments of consciousness do you use in your clinical or research practice? (may choose more than one): H2O (PET) (FDG) PET Resting-state fMRI Active fMRI Standard EEG Quantitative analysis Qualitative analysis Sleep EEG Quantitative analysis Qualitative analysis High-density EEG Quantitative analysis Qualitative analysis Somatosensory-Evoked Potentials Brain stem-Evoked Potentials Event-Related Potentials Transcranial Magnetic Stimulation (TMS)-EEG Brain–Computer Interface (BCI) Other (specify) - If you use neuroimaging, what kind of paradigm do you perform? (may choose more than one): Resting state Passive sensory stimulation Active tasks (may choose more than one) None of the above
“Standardized clinical evaluation, EEG-based techniques and functional neuroimaging should be integrated for multimodal evaluation of patients with DoC.”	“In situations where there is continued ambiguity regarding evidence of conscious awareness despite serial neurobehavioral assessments, or where confounders to a valid clinical diagnostic assessment are identified, clinicians may use multimodal evaluations incorporating specialized functional imaging or electrophysiologic studies to assess for evidence of awareness not identified on neurobehavioral assessment that might prompt consideration of an alternate diagnosis”	- Is it possible in your center (e.g., in terms of technology and expertise) to integrate expert clinical evaluation (i.e., from > 10 years experts), EEG-based techniques, and/or functional neuroimaging for the evaluation of patients with DoCs? - How often are the approaches above combined? - What is the main challenge in the implementation of a multimodal assessment of consciousness in your program with regards to utilization of high-density EEG, PET, and fMRI?
**Families counseling**		
	“Counsel families that for adults, MCS (vs vegetative state [VS]/ unresponsive wakefulness syndrome [UWS]) and traumatic (vs nontraumatic) etiology are associated with more favorable outcomes”. The AAN Guidelines also say that “when prognosis is poor, long-term care must be discussed”	- Do you or your team regularly counsel families or patient’s representatives/caregivers about the patient’s diagnosis, prognosis, and possible long-term care options? - If yes, when do you provide said counseling? - Has the COVID-19 pandemic changed your practice in this regard?
	“Clinicians must identify patient and family preferences early and throughout provision of care to help guide the decision-making process for persons with prolonged DoC.”	- Do you attempt to identify patient and family treatment preferences (e.g., therapeutic and palliative interventions) soon after admission? - Do you or your team inform families about the limitations of existing evidence concerning currently employed (standard) treatment and/or non-validated (e.g., experimental) treatment effectiveness and the related potential risks and harms?
**Prognosis and rehabilitation**		
	“Structural MRI, SPECT, and the Coma Recovery Scale-Revised (CRS-R) can assist prognostication in adults”	- What methods do you use for assisting with outcome prognostication? - Does your team provide evidence-based prognosis information to family members of patients with DoC
	“Care for patients with prolonged DoC may benefit from a team of multidisciplinary rehabilitation specialists”	- Do you have a specific DoC rehabilitation program in your center? - Is the rehabilitation program regularly updated on the basis of repeated assessments of consciousness and/or of functional disability?
**Pain**		
“The NCS-R [Nociceptive Coma Scale Revised] is considered for regular monitoring of signs of discomfort”	“Pain always should be assessed and treated […] and evidence supporting treatment approaches discussed”. They also say that “Clinicians should assess individuals with a DoC for evidence of pain or suffering and should treat when there is reasonable cause to suspect that the patient is experiencing pain […], regardless of level of consciousness. Clinicians should counsel families that there is uncertainty regarding the degree of pain and suffering that may be experienced by patients with a DoC […].”	- Do you specifically assess pain in patients with DoCs? If yes, how? - Would you treat pain if you are unsure about the patient’s level of consciousness based on bedside assessment? - Should pain be treated (e.g., through use of pain medications) regardless of the level of residual consciousness? If yes, how? - Do you counsel families about the difficulty of detecting pain in patients with DoCs?
**Collaboration with other centers**		
Multicenter collaborations are needed		- Do you work collaboratively with other DoC centers/experts? If yes, in what context? Clinical Research Both
**Nosology**		
	The AAN Guidelines say that “Recent evidence indicates that the term chronic VS/UWS should replace permanent VS, with duration specified”	- Should the term permanent VS or UWS be replaced with the "VS/UWS and its specific duration"?

The final version of the survey, which was anonymous and took about 15 min to complete, included 48 items divided in 7 sections with different numbers of questions: respondent demographics (*n* = 6), diagnosis (*n* = 18), families counseling (*n* = 11), prognosis and rehabilitation (*n* = 4), pain (*n* = 6), collaborations with other centers (*n* = 2), and nosology (*n* = 1) (see Table 1). Most of the items consisted of closed-ended questions with single- or multiple-choice answers, but some open-ended questions were also included (see the Supplementary Materials for the complete survey).

The survey was launched on February the 1^st^ 2022 through the online platform Survey Monkey (SurveyMonkey Inc., SanMateo, California, USA; www.surveymonkey.com), and was available for 1 month. The invitation was sent to the IBIA mailing list as well as to other relevant international and national professional associations (i.e., EAN, ACRM, Japan Coma Society, North America Brain Injury Society, European Brain Injury Society, International Neurotrauma Society, Curing Coma Campaign, Italian Society of Neurorehabilitation, Italian Society of Physical and Rehabilitation Medicine, European Federation of Neurological Associations, Federation of European Neuroscience Societies, Swedish Neurological Society, Swedish Society for Anesthesia and Intensive Care, World Federation of Neurorehabilitation, National Neurotrauma Society, Neurocritical Care Society, Society of Neurological Surgeons, American Association of Neurological Surgeons, Canadian Neurological Sciences Federation, Asian Australasian Society of Neurological Surgeons, Indian Federation of Neurorehabilitation, Indian Academy of Neurology, India National Institute of Mental Health and Neurosciences, Neurological Society of India, Korean Association of Rehabilitation Medicine, Korean Neurological Association, Korean Neurosurgical Society, Taiwan Academy of Physical Medicine and Rehabilitation, Hong Kong Association of Rehabilitation Medicine, The Hong Kong Neurosurgical Society, Bangladesh Association of Physical Medicine and Rehabilitation, Malaysia Society of Rehabilitation Physicians, Malaysian Society of Neurosciences, Japan Neurosurgical Society, African Academy of Neurology, Society of Neuroscientists of Africa, Society of Neurosurgeons of South Africa, and Neurosurgical Society of Australasia) and to personal connections of the collaborators.

Data were exported to Excel (Microsoft, WA, United States) and checked to exclude any duplicates.

The study has been approved by the Ethics Committee of the Faculty of Medicine of the University Hospital of Liège (Belgium; reference 2019/235).

### Data analyses

Responses and omissions were calculated for each question. Not all respondents answered all the questions, and results are, therefore, reported on the basis of total responses for each question. Responses toward the end of the questionnaire were most likely to be missing. Missing data were handled by pairwise deletion. The proportion of responses for all possible answers to closed-ended questions were calculated, whereas responses to open-ended questions were analyzed qualitatively.

For the purposes of the analysis, data were grouped as a function of respondent demographics into three geographical areas (Europe, North America, and APA which included Asia–Pacific and Africa), four clinical settings (i. Intensive Care Units, ICU; ii. Intensive Specialized Rehabilitative Units, ISRU; iii. Specialized Care Facilities for chronic patients, SCF; iv. Other), and two levels of expertise (less than 10 years and more than 10 years). Percentages of responses for each item were compared between respondents in diverse groups through *χ*^2^ tests. The level of significance was set at 0.05. All analyses were performed using IBM SPSS v.25 (IBM Corp., Armonk, New York, USA).

## Results

**Fig. 1:**
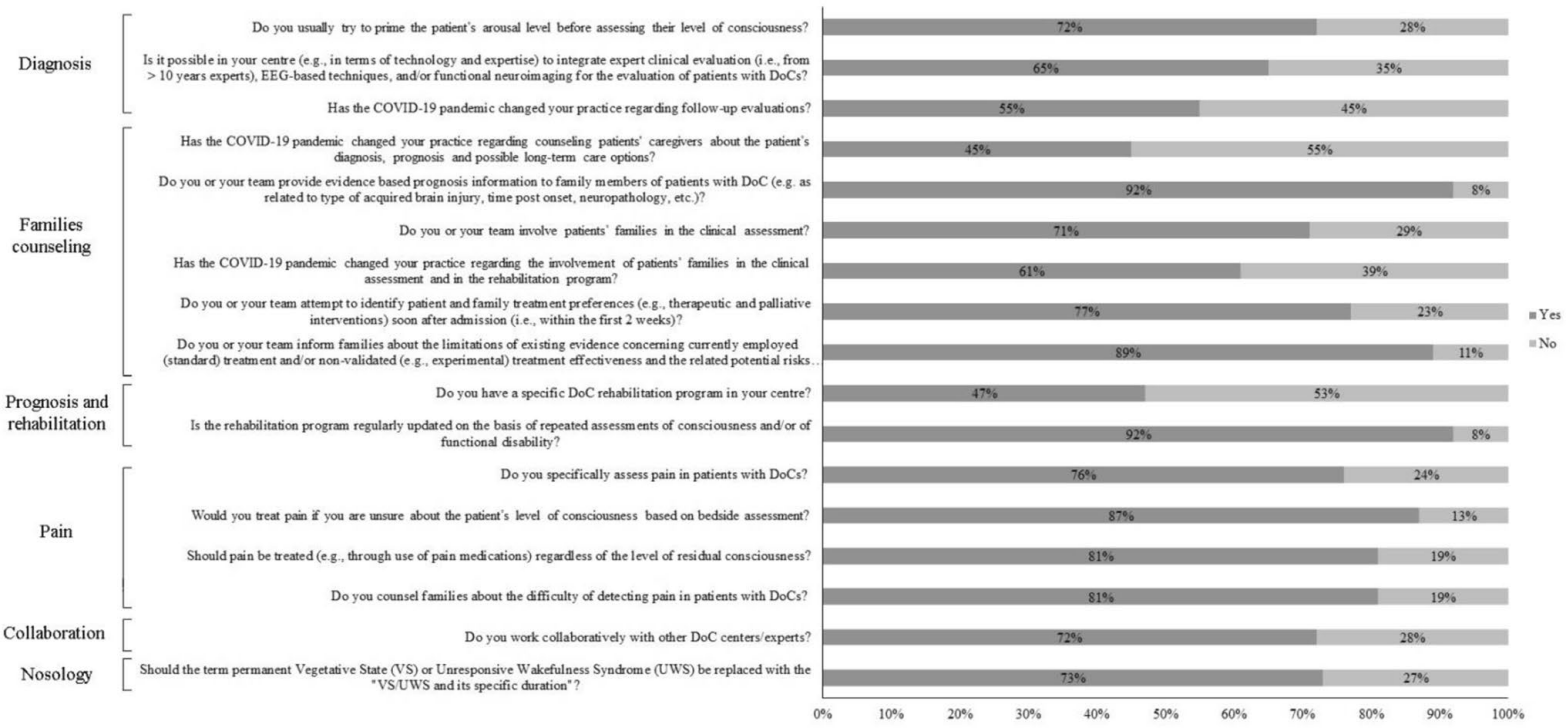
Main results of the survey

We report below main results from the survey according to the sections described above. If not reported, there are no significant differences among subgroups. Figure 1 summarizes the main results. For more detailed information see Supplementary Table 1.

### Respondent demographics

**Fig. 2:**
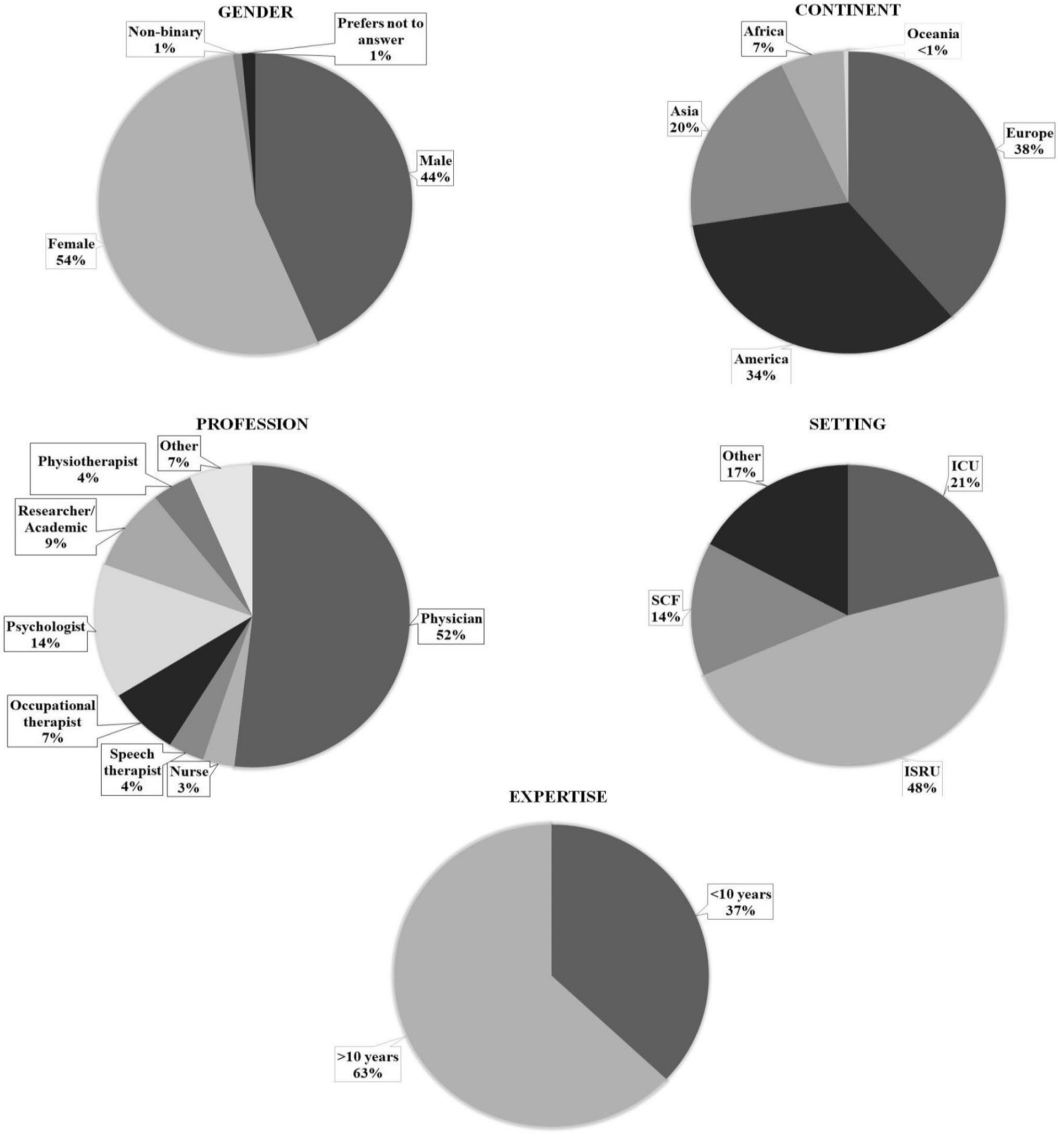
Respondents’ demographics (*n* = 216). *SCF* Specialized Care Facility for chronic patients, *ICU* Intensive care unit, *ISRU* intensive specialized rehabilitation unit

Two hundred and sixteen professionals participated in the survey (*n* = 117/216; 54% females; 95/216; 44% males; mean age = 50 ± 13 years; see Fig. 2). The majority of respondents were health professionals (*n* = 189/216; 87%), and the remaining respondents were academicians. Among the participating professionals, the majority were physicians (*n* = 112/216; 52%). The most represented clinical setting was Intensive Specialized Rehabilitation Unit (ISRU) (*n* = 103/216; 48%). The majority of respondents had more than 10 years of experience (*n* = 136/216; 63%). The geographic location of respondents quite varied: Europe (*n* = 83/216; 38%), America (*n* = 73/216; 34%), Asia–Pacific (*n* = 45/216; 21%), and Africa (*n* = 15/216; 7%), for a total of 40 countries.

### Diagnosis

#### Clinical assessment

The most used diagnostic tool by respondents was bedside behavioral examination with standardized tools (*n* = 154/196; 79%), followed by neurophysiological evaluation (i.e., EEG; *n* = 105/196; 54%) and structural neuroimaging (*n* = 101/196; 52%).

Bedside standardized tools and neurophysiological evaluations were used most frequently in Europe (*n* = 71/80; 89% and 53/80; 66%, respectively) compared to the rest of the world (*n* = 35/55; 64%; *p* =  < 0.001) and to America (*n* = 23/61; 38%; *p* = 0.001), respectively. A neurophysiological evaluation was also more frequently utilized by respondents with longer durations of clinical experience (*n* = 76/126; 60%; *p* = 0.011), as compared to respondents with less than 10 years of expertise (*n* = 29/70; 41%).

The majority of respondents (*n* = 79/131; 60%) stated that they performed repeated behavioral assessments, with a lower rate in Europe (*n* = 25/57; 44%) than in North America (*n* = 29/41; 71%; *p* = 0.028) and APA (*n* = 25/33; 76%; *p* = 0.013).

Concerning how often the respondents repeated the clinical assessment, the majority of them (*n* = 82/131; 63%) answered that it depended on the patient’s condition, while 35% (*n* = 46/131) of them repeated assessment regularly and equally for all patients. The frequency of repeated assessment was highly variable: weekly or twice weekly (*n* = 17/131; 13%), daily (*n* = 13/131; 10%), or depending upon the patient’s condition (*n* = 15/131; 11%).

As recommended by the US guidelines, the majority of respondents (*n* = 120/166; 72%) declared to usually try to prime the patient’s arousal level before assessing their level of consciousness, mainly through auditory stimulation (*n* = 95/114; 83%) and tactile stimulation (*n* = 90/114; 79%).

The majority of respondents (*n* = 149/196; 76%) declared to always observe the patient prior to actually doing their hands-on clinical assessment. The main reason given by respondents was to observe spontaneous behavior (*n* = 33/196; 17%).

#### Diagnostic tools and multimodal assessment

The most frequently utilized diagnostic tools endorsed by respondents were the CRS-R (*n* = 98/131; 75%) and GCS (*n* = 83/131; 63%). The CRS-R is mostly used in ISRU settings (*n* = 60/69; 87%; ICU: *n* = 22/32; 69%; *p* = 0.029 and SCF: *n* = 12/21; 57%; *p* = 0.003) and geographically most commonly used in Europe (*n* = 47/57; 82%) and North America (*n* = 33/40; 82%) compared with APA (*n* = 18/33; 54%; *p* = 0.004 and *p* = 0.010, respectively).

The most used technological assessments were quantitative (*n* = 71/131; 54%) and qualitative (*n* = 61/131; 47%) analysis of standard EEG, Somatosensory-Evoked Potentials (SEPs; *n* = 56/131; 43%), and brainstem EP (*n* = 50/131; 38%). SEPs were recorded more frequently in Europe (*n* = 33/57; 58%) than in North America (*n* = 11/41; 27%; *p* = 0.002) and less so in APA (*n* = 12/33; 36%; *p* = 0.049), whereas brainstem EPs were more frequently utilized in Europe (*n* = 28/57; 49%) compared with North America (*n* = 9/41; 22%; *p* = 0.006) and by respondents with more years of clinical practice (*n* = 40/90; 44% vs. 10/41; 24%; *p* = 0.028).

Among respondents who use neuroimaging techniques, the most performed paradigms were resting-state paradigm (*n* = 74/131; 56%) and active tasks (*n* = 24/131; 18%). Resting-state and passive paradigms were performed more frequently in APA (*n* = 26/33; 79% and 11/33; 33%, respectively) than in Europe (resting state:* n* = 32/57; 56%; *p* = 0.031 and passive: *n* = 5/57; 9%; *p* = 0.003) and in North America (resting state:* n* = 16/41; 39%; *p* = 0.001 and passive: *n* = 2/41; 5%; *p* = 0.001). In general, passive paradigms resulted to be more used by respondents with fewer years of clinical practice (*n* = 10/41; 24% vs. 8/90; 89%; *p* = 0.017).

The majority of respondents (*n* = 102/158; 65%) declared that it is possible (e.g., in terms of technology and expertise) to integrate clinical evaluation based on experience (i.e., from > 10 years of practice), EEG-based techniques, and/or functional neuroimaging for the evaluation of patients with DoCs. Around 54% (*n* = 85/158) of the respondents declared that the integration of the aforementioned methods was commonly utilized, whereas 26% (*n* = 41/158) opined that it was not a common utilized practice. Multimodal assessment was more frequent in ICU (*n* = 31/39; 79%) than in ISRU (*n* = 48/81; 59%; *p* = 0.029) and less frequently endorsed for SCF (*n* = 12/23; 52%; *p* = 0.024). The main reasons for not performing an integrated assessment process were noted to be lack of technology (*n* = 41/158; 26%), lack of expertise/skilled personnel (*n* = 20/158; 13%), and prohibitive costs (*n* = 11/158; 7%).

### Family counseling and needs

The majority of respondents answered that they or their team regularly counseled families or patient’s representatives/caregivers about the patient’s diagnosis, prognosis, and possible long-term care options (*n* = 92/107; 86%), mainly during hospital stay (*n* = 86/93; 92%), and more frequently when in ISRU (*n* = 55/57; 96%) than when in ICU (*n* = 18/25; 72%; *p* = 0.004) or in SCF (*n* = 12/16; 75%; *p* = 0.006).

The majority of respondents reported that they attempt to identify patient and family treatment preferences (e.g., therapeutic and palliative interventions) soon after admission (*n* = 84/109; 77%; more frequently in ICU (*n* = 24/25; 96%) than in ISRU (*n* = 40/57; 70%; *p* = 0.009)). Additionally, the vast majority (*n* = 97/109; 89%) endorsed informing families about the limitations of existing evidence concerning standard treatment and/or non-validated/experimental treatment effectiveness and their related risks.

### Prognosis and rehabilitation

Almost all the respondents (*n* = 100/109; 92%) answered that their team provided evidence-based prognostic information to patients’ families. The factors commonly reported for assisting with outcome prognostication were: etiology of brain injury (*n* = 87/109; 80%), medical complications (*n* = 86/109; 79%), medical history (*n* = 84/109; 77%), age (*n* = 82/109; 75%), duration of DoCs (*n* = 80/109; 73%), behavioral assessment results (*n* = 75/109; 69%, mainly through CRS-R, 13%), structural MRI (*n* = 73/109; 67%), and standard EEG (*n* = 63/109; 58%).

The etiology of the brain injury (*p* = 0.001) and medical complications (*p* = 0.008) were taken into account as prognostic indices more frequently in ISRU (*n* = 51/57; 89% and 50/57; 88%, respectively) than in SCF (*n* = 9/17; 53% and 10/17; 59%, respectively), and the duration of DoC was more often used in ISRU (*n* = 49/57; 86%) than in SCF (*n* = 10/17; 59%; *p* = 0.015) and ICU (*n* = 16/25; 64%; *p* = 0.024). Conversely, structural MRI was used more in the ICU (*n* = 21/25; 84%) than in SCF (*n* = 7/17; 41%; *p* = 0.004). Medical complications and duration of DoCs were also used more frequently as prognostic indices by respondents with longer time in clinical practice (medical complications: *n* = 63/74; 85% vs. *n* = 23/34; 68%; *p* = 0.036 and duration of DoCs: *n* = 59/74; 80% vs. 21/34; 62%; *p* = 0.048), and in America more than in the rest of the World (medical complications: *n* = 32/35; 91% vs. *n* = 17/26; 65%; *p* = 0.011 and duration of DoCs: *n* = 28/35; 80% vs. 13/26; 50%; *p* = 0.014). The duration of DoCs was also used as a prognostic factor in Europe (*n* = 39/47; 83%) more so than in APA (*n* = 13/26; 50%; *p* = 0.003).

The majority of respondents (*n* = 58/109; 53%) reported that they did not have a specific rehabilitation program in their center for persons with DoCs (especially in ICU, *n* = 5/25; 20% vs. IRU, 33/57; 58%; *p* = 0.002). Eighty-four percent (*n* = 43/51) of the 47% of respondents who do have a specific DoC rehabilitation program declare that it is aimed for both consciousness and functional recovery.

### Pain

The majority of respondents noted that they assessed for pain (*n* = 83/109; 76%), mainly through clinical assessment (*n* = 49/83; 59%) followed by NCS-R (*n* = 39/83; 47%). Clinical assessment of pain was more frequently endorsed in North America (*n* = 20/25; 80%) and in APA (*n* = 14/20; 70%) in comparison to Europe (*n* = 15/38; 39%; *p* = 0.002 and *p* = 0.027, respectively), where the NCS-R is more frequently used than in America (*n* = 23/38; 60% vs 6/25; 24%; *p* = 0.004). Also, the majority, especially in Europe (*n* = 46/48; 96%) vs. America (*n* = 28/34; 82%; *p* = 0.043) and the rest of the world (*n* = 20/26; 77%; *p* = 0.012), noted that they would treat pain even if unsure about the patient’s level of consciousness based on bedside assessment (*n* = 94/108; 87%), and were of the opinion that pain should be treated through use of pain medications regardless of the level of residual consciousness (*n* = 87/108; 81%). This last statement was endorsed more frequently by professionals in ICU (*n* = 21/25; 84%) and ISRU (*n* = 49/56; 87% vs. SCF: 10/18; 55%; *p* = 0.040 and *p* = 0.003, respectively) and among those in practice longer (*n* = 23/34; 68% vs. 64/74; 86%; *p* = 0.022). The majority noted that they counseled families about the difficulty of detecting pain in patients with DoCs (*n* = 87/108; 81%).

### Collaboration

Seventy-two percent (*n* = 78/108) of respondents work collaboratively with other centers and experts working with patients with DoCs: 32% (*n* = 25/78) for clinical activity, 17% (*n* = 13/78) for research activity, and 51% (*n* = 40/78) for both.

### Nosology

The majority of respondents opined that the term permanent VS or UWS should be replaced with the phrase "VS/UWS and its specific duration" (*n* = 77/106; 73%).

### Impact of COVID-19

For the majority of the respondents, the COVID-19 pandemic has changed the practice in regard to the frequency of follow-ups (*n* = 71/129; 55%), although with a lower impact in North America (*n* = 16/40; 40%) than in APA (*n* = 22/32; 84%; *p* = 0.015). The change occurred mainly because of the lack of physical interaction (*n* = 41/129; 32%), reduced follow-ups (*n* = 23/108; 18%), and less possibility for hospitalization (*n* = 14/129; 11%). The COVID-19 pandemic changed the involvement of families in 61% (*n* = 66/109) of cases, always because of the restrictions to physical interaction due to infection control constraints. Occasionally, families were involved through remote connections (*n* = 7/109; 7%).

## Discussion

Starting from the guidelines’ recommendations, the aim of the survey was to explore the clinicians’ actual clinical practice and their opinions about the reliability, relevance, and applicability of the guidelines in different contexts. Based on some differences between the EU and US guidelines (e.g., US guidelines give specific attention to pediatric patients and to patients’ families counseling) and the actual practice declared by the respondents, the present survey indicates that some recommendations are less implemented than others (see Table 2 for the most relevant).

**Table 2:** Most and least implemented recommendations

Topic	Most implemented recommendations	Least implemented recommendations
**Diagnosis**	· Increasing arousal before assessing residual consciousness · Using CRS-R for behavioral assessment · Observing spontaneous motor behavior before assessing consciousness	· Repeating clinical assessment of consciousness. Importantly, there is no consistency among respondents about the modality/frequency · Integrating bedside behavioral examination with technological assessment · Implementing a multimodal diagnostic assessment
**Prognosis**	· Providing evidence-based prognosis information to family members · Counseling families, identify patients’ and their families’ preferences, and inform families about the limitations of actual diagnostic, prognostic, and therapeutic approaches	
**Pain**	· Specifically assessing it · Counseling family members about the difficulty to assess pain	Using NCS-R
**Collaboration**	· Collaborating with other centers	
**Nosology**	· Replacing the term permanent or chronic VS/UWS with the "VS/UWS and its specific duration"	

Among other things, it emerges that the repetition of clinical assessment is not consistently implemented. In fact, the majority of respondents reported that the frequency of repeating the clinical assessments depends on the patient’s condition. While, on the one hand, this is reasonable, on the other hand, it raises the need for a better standardization of the assessment procedure.

The same about the multimodal diagnostic strategy recommended by both the guidelines. The integration of bedside and technological assessment is possible for the majority of respondents, but it still faces important challenges, including lack of sufficient expertise, difficulties in accessing necessary technology, lack of time, and costs. All these factors need to be adequately accounted for to make the recommendations more effective and impactful.

At the clinical level, in addition to the results above, it is interesting to highlight that the COVID-19 pandemics impacted two dimensions in particular of the care of patients with DoCs: follow-ups and family´s involvement. In both cases, the participants to the survey reported a reduction of visit frequency primarily due to restrictions to physical interaction. This point seems to fit to common radical changes in the organization and in the operating methods in rehabilitation settings during COVID waves [15].

In addition to their clinical relevance, the above-mentioned results also have ethical implication of significance. The ethical reflection about patients with DoCs is an urgent need that emanates from the many recent scientific and technological advancements in the field [16–19].

The fundamental need behind both the guidelines is to ensure a more precise diagnosis, to avoid the misdiagnosis of patients with covert awareness, and to allow a more reliable prognosis. Especially, the improvement of present diagnostic accuracy is indispensable, in the light of recent research suggesting a high number of patients considered as UWS at bedside but showing residual conscious functioning (e.g., MCS*) [20].

Against this background, three results from the survey deserve specific ethical attention (see Table 3): the limited implementation of multimodal diagnosis; the actual practice about pain detection and treatment; and the impact of COVID-19 pandemics.

**Table 3:** Main ethical challenges resulting from the survey

Ethical challenge
Limited implementation of **multimodal diagnosis** due to lack of technology, lack of skilled personnel, and prohibitive costs, among other things
Balancing a precautionary approach about **pain perception** by patients with DoCs **and its treatment**, and the need for avoiding the risk of impacting ethically relevant capacities (e.g., autonomy and self-determination) due to analgesic treatment
Safeguarding a sufficient involvement of patients’ representatives despite the **restrictions imposed by COVID-19 pandemics**

As seen above, the main point of both the guidelines is supporting and eventually improving the multimodal assessment of residual consciousness in patients with pDoCs. According to our survey, the combination of behavioral examination and technological assessment is possible in only about half of the cases. This is likely caused by a number of factors, including lack of technology, lack of skilled personnel, and excessive costs. If multimodal assessment should be the gold standard for making an accurate diagnosis, this approach could be time-consuming and requires specific expertise. The obstacles identified in our survey require a strategy for a more equal and fair distribution of resources, which may be pursued also by new technological means. For instance, highly specialized central hubs of DoCs expertise may be created or further enhanced, to be consulted also virtually when needed [21].

Regarding pain, the majority of clinicians think that it should be treated regardless of the level of residual consciousness. This may be problematic, since the use of pain-killers might raise the risk of reducing residual awareness, impacting ethically relevant capacities like autonomy and self-determination [22–24]. Therefore, it is necessary to further explore this issue (e.g., to keep investing in research aimed at minimizing the side effects of analgesic treatments) to get a more consistent and ethically balanced view on this point.

Finally, the impact of COVID-19 pandemics on the actual clinical care of DoCs deserves specific ethical attention, particularly the limitation of interaction with the patients´ representatives that it caused. Overall, the actual clinical practice as reflected in our survey results in line with the recommendations to involve the patients’ representatives (e.g., their families) through counseling services. This is a good practice that should be emphasized and further promoted. In fact, it is worth to better involve patients’ caregivers in the clinical treatment for avoiding medical paternalism, therapeutic nihilism, and misleading communication leading to false hope [25] and in the behavioral assessment, as they are the most salient source of subjective stimulation for eliciting intentional conscious behaviors [26, 27]. The COVID-19 pandemics may be taken as a case study to think about potential drawbacks of present involvement of patients’ representatives, to plan corrective measures to actual practice and preventive strategies for possible future similar scenarios.

The survey also showed a number of good practices among professionals working with patients with DoCs, notably elaborating evidence-based prognosis, counseling families about the limitation of prognosis and pain detection as well as collaborating with other centers. These points should be considered by all clinicians working with these patients and promoted to facilitate more efficient and appropriate clinical treatment.

The present study has a number of limitations: the first one is the relatively small number of respondents and the selection bias as the majority of respondents are affiliated to scientific societies involved in the field of DoCs. Moreover, we found that a certain percentage of respondents did not answer all questions. This may have raised a possible selection bias threatening the analyses of some answers, which could reflect the judgments of a particular “more-committed” sub-sample of respondents. These limitations could hamper the generalization of results. However, it is worth underlining that the responses of this selected sample of participants specifically highlight the need for optimizing the strategies to implement EU and US guidelines even in highly specialized and informed clinicians, as members of scientific societies likely are. Another limitation is the use of closed question formats. The use of such format has the advantage to quantitatively assess a question, but the inherent limitation is that this constrains the respondents to a limited list of response suggestions. Missing data (up to 50% in the last part of the survey with respect to respondents that started the survey) are another limitation and future online surveys should make the answers mandatory. As reported in Supplementary Table 1, the diagnosis section has the highest number of respondents while the counseling section has the lowest number of respondents. For all the questions combined, the average number of respondents is 113/216, with no significant differences between countries, expertise, and settings.

### Final suggestions and future perspectives

Notwithstanding its limitations, the following regulatory needs emerge from the present analysis:

- Elaborating a strategy for assessing the main factors that limit the integration of clinical evaluation, EEG-based techniques, and/or functional neuroimaging, specifically lack of technology, lack of expert/skilled personnel, and high costs. For instance, the creation or the enhancement of specialized hubs of DoCs expertise, at both national and international levels, may be a fruitful strategy to implement [21].
- Elaborating a strategy for improving a multimodal diagnostic approach, particularly the use of neurotechnology (e.g., elaborating clear and consistent recommendations about when to use it, and introducing standards for the interpretation of emerging results) [28, 29]. A promising model to further explore is, for instance, a structured approach to DoCs at the national level [30], possibly complementing it with relevant international collaborations.
- Developing a more robust, evidence-based recommendation on the frequency of the assessment of consciousness to better standardize clinical practice, for instance through the use of a simplified behavioral assessment [31, 32].
- Identifying the underlying causes of the lack of specific and multidisciplinary rehabilitation programs, and elaborating concrete strategies and action plans for including them in the ordinary care of patients with DoCs, not only in IRU [33].
- Elaborating clear and specific recommendations about the detection of pain, possibly taking inspiration from relevant ongoing research, also by means of caregivers reports, as well as about the treatment of pain and discomfort in patients with DoCs, particularly for minimizing any possible risk of side effects impacting the level of residual awareness [24, 34–37].
- Investigating further whether and how the COVID-19 pandemics impacted the quality of care eventually provided to patients, and thinking how to avoid this negative impact if further restrictions will be needed [15, 38].
- Investigating how to promote a better in-person and/or remote interaction between patients’ families and clinicians and between patients and their families (e.g., the use of telephonic and virtual appointments/interactions), identifying the factors leading to mismatches between the caregivers’ and the clinicians’ perspectives [39].

## Conclusion

A survey among professionals working with patients with DoCs revealed that while some recommendations from EU and US guidelines are being followed, others are not and/or may require more honing/specificity to enhance their clinical utility. Particularly, enhancing a multimodal diagnostic approach, promoting a consistent treatment of pain and discomfort, and elaborating strategies for facing restrictions to the interaction with the patients’ representatives similar to those caused by the COVID-19 pandemics emerge among the most pressing needs. Further efforts to facilitate implementation of the guidelines is of utmost importance to clinical practice along with ongoing efforts at updating guidelines based on current evidence-based medicine with an ultimate goal of developing international consensus.

### Supplementary Information

Below is the link to the electronic supplementary material.

Supplementary file1 (PPTX 1380 KB)

Supplementary file2 (DOCX 61 KB)

## Data Availability

The raw data that support the findings of this study are available in OSF with the identifier 10.17605/OSF.IO/AYJ9X.
