# Correction to: International survey on the implementation of the European and American guidelines on disorders of consciousness

**DOI:** 10.1007/s00415-023-12083-5

**Published:** 2023-11-22

**Authors:** Michele Farisco, Rita Formisano, Olivia Gosseries, Yoko Kato, Shigeki Koboyashi, Steven Laureys, Nicolas Lejeune, Charlotte Martial, Amal Matar, Ann-Marie Morrisey, Caroline Schnakers, Maidinamu Yakufujiang, Tomohiro Yamaki, Vigneswaran Veeramuthu, Matteo Zandalasini, Nathan Zasler, Alfonso Magliacano, Anna Estraneo, IBIA Special Interest Group on DoCs

**Affiliations:** 1 Centre for Research Ethics and Bioethics, Uppsala University, Uppsala, Sweden; 2 Biogem, Biology and Molecular Genetics Research Institute, Ariano Irpino, AV Italy; 3 IRCCS Santa Lucia Foundation, Rome, Italy; 4 Coma Science Group, GIGA-Consciousness, University of Liège, Liège, Belgium; 5 Centre du Cerveau, University Hospital of Liège, Liège, Belgium; 6 Department of Neurosurgery, Fujita Health University Bantane Hospital, Nagoya, Aichi Japan; 7 Division of Neurosurgery, Rehabilitation Center for Traumatic Apallics Chiba, National Agency for Automotive Safety and Victims’ Aid, 3-30-1 Isobe, Mihamaku, Chibashi, Chiba 261-0012 Japan; 8 CERVO Brain Research Center, University of Laval, Québec, QC Canada; 9 CHN William Lennox, Ottignies-Louvain-La Neuve, Belgium; 10 Institute of NeuroScienceUCLouvain, Ottignies-Louvain-La Neuve, Belgium; 11 School of Allied Health, Faculty of Education and Health Sciences, Ageing Research Centre, Health Research Institute, University of Limerick, Limerick, Ireland; 12 Research Institute, Casa Colina Hospital and Centers for Healthcare, Pomona, CA USA; 13 Thomson Hospital Kota Damansara, Petaling Jaya, Selangor Malaysia; 14 Unità Spinale, Neuroriabilitazione E Medicina Riabilitativa Intensiva, Dipartimento Di Medicina Riabilitativa, Azienda USL Di Piacenza, Piacenza, Italy; 15 Concussion Care Centre of Virginia, LTD, Henrico, VA 23233 USA; 16 Department of Physical Medicine and Rehabilitation, Virginia Commonwealth University, Richmond, VA 23298 USA; 17 IRCCS Fondazione Don Carlo Gnocchi ONLUS, Florence, Italy

**Correction to: Journal of Neurology** 10.1007/s00415-023-11956-z

In the original version of this article, the affiliation detail for author Anna Estraneo was incorrectly given as

IRCCS Fondazione Don Carlo Gnocchi ONLUS, Florence and Sant’Angelo dei Lombardi, AV, Italy

but should have been:

IRCCS Fondazione Don Carlo Gnocchi ONLUS, Florence, Italy

