# Supplementary material for: International survey on the implementation of the European and American guidelines on disorders of consciousness
Source: J Neurol. 2023 Sep 23;271(1):395–407. doi: 10.1007/s00415-023-11956-z (PMC10770208; doi:10.1007/s00415-023-11956-z)

## Slide 1
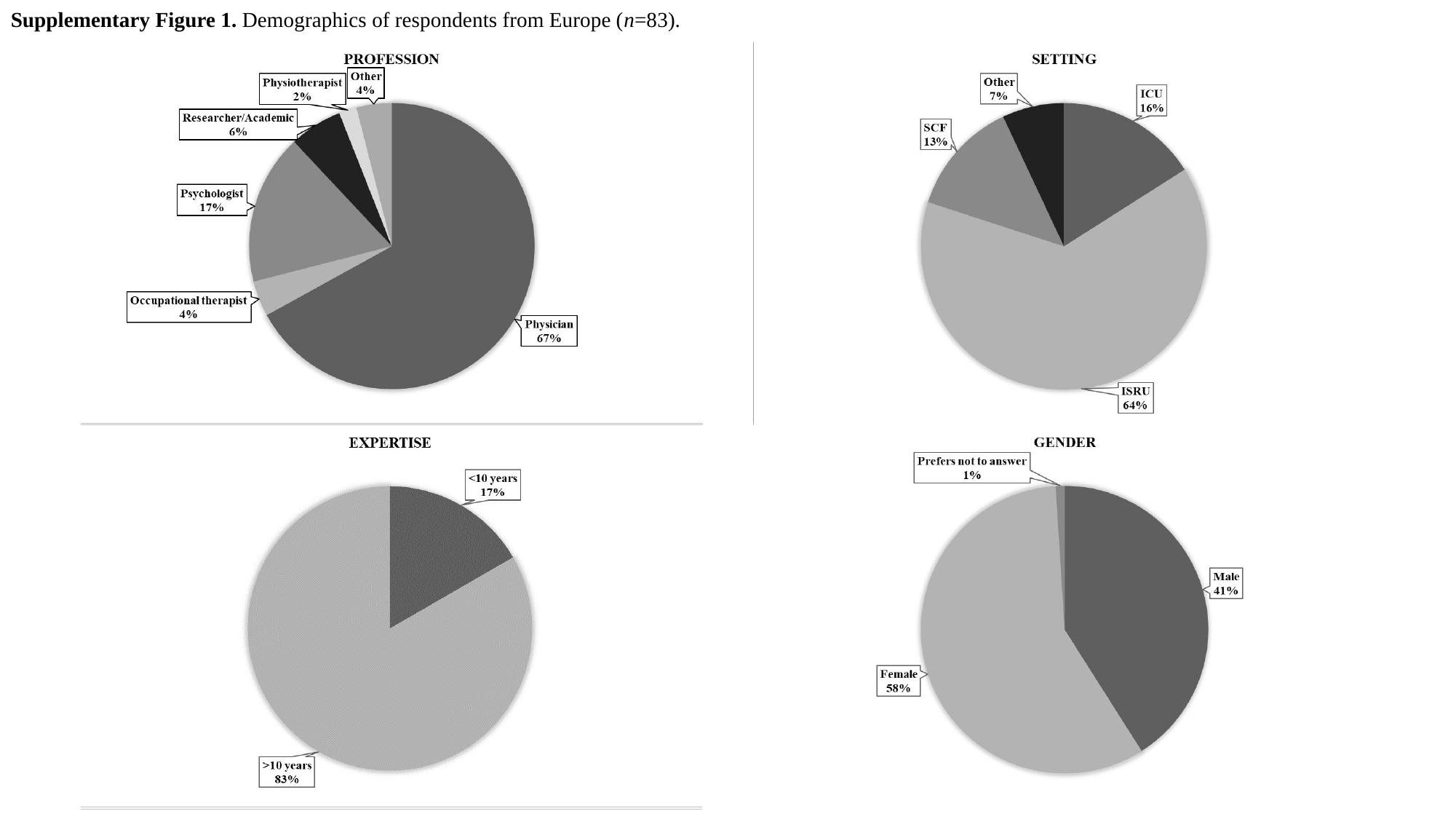

Supplementary Figure 1. Demographics of respondents from Europe (n=83).

## Slide 2
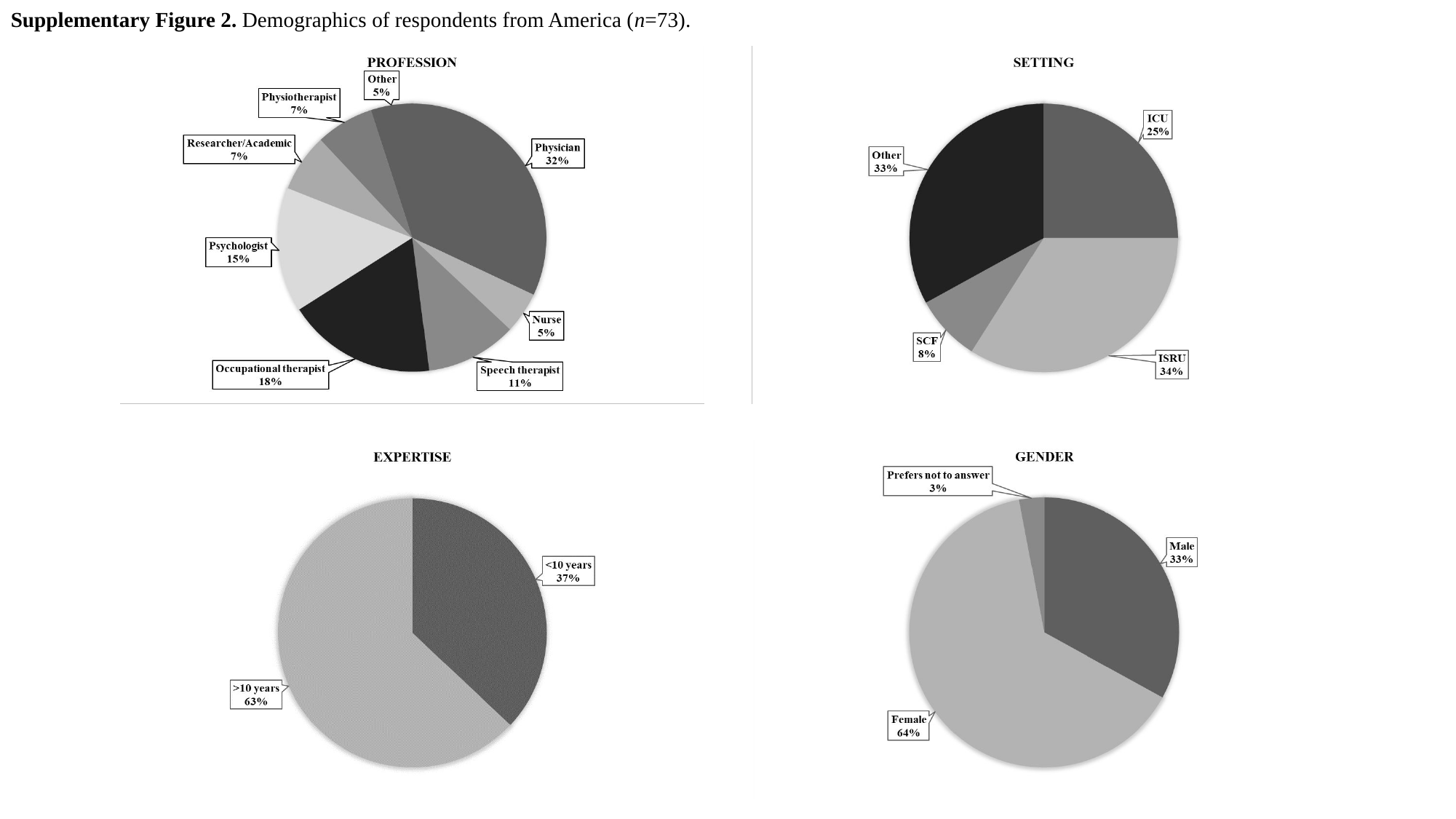

Supplementary Figure 2. Demographics of respondents from America (n=73).

## Slide 3
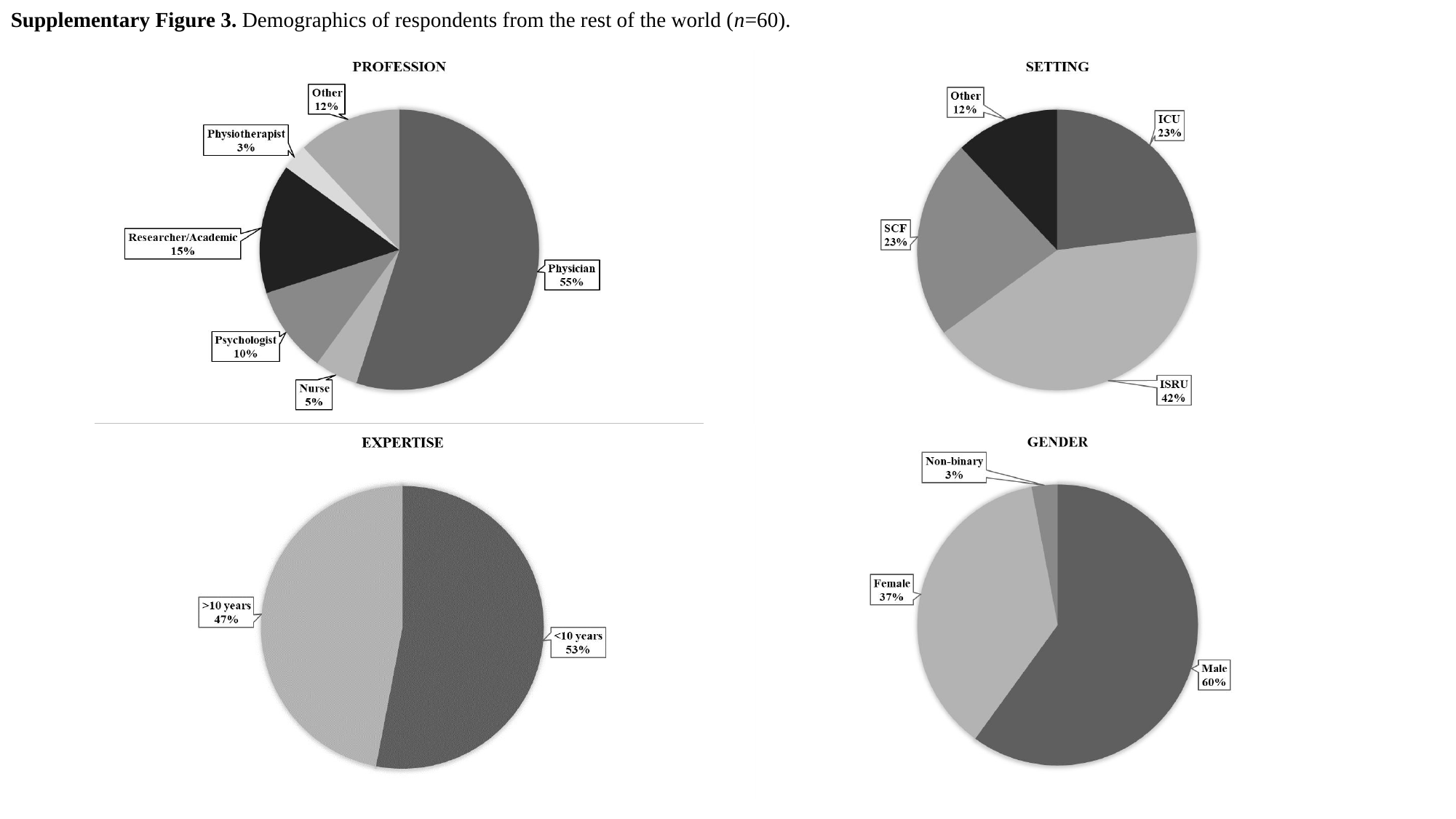

Supplementary Figure 3. Demographics of respondents from the rest of the world (n=60).

## Slide 4
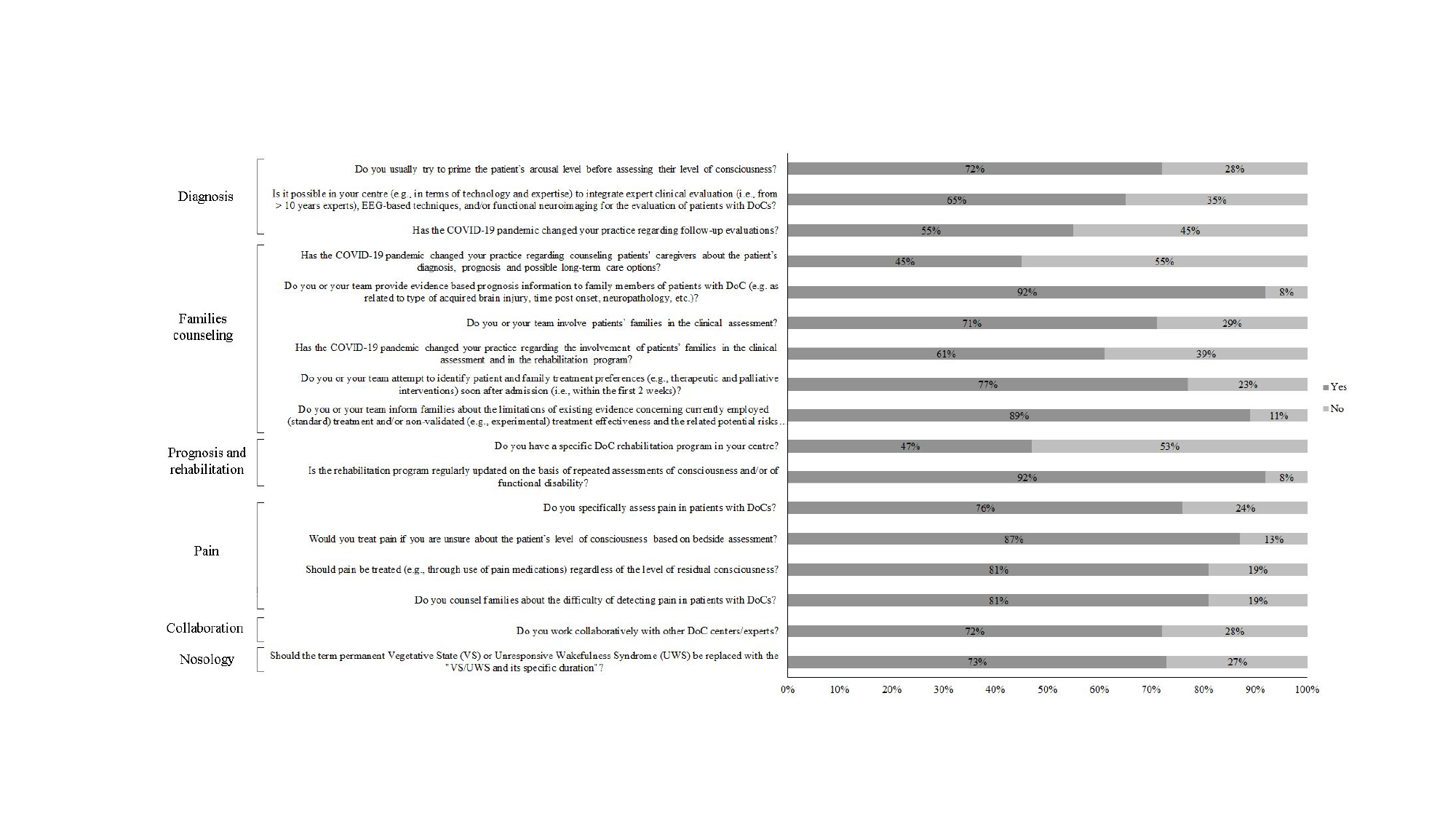

Supplement: Supplementary file 1 — Supplementary file1 (PPTX 1380 KB) [file 415_2023_11956_MOESM1_ESM.pptx]
