# Supplementary material for: International survey on the implementation of the European and American guidelines on disorders of consciousness
Source: J Neurol. 2023 Sep 23;271(1):395–407. doi: 10.1007/s00415-023-11956-z (PMC10770208; doi:10.1007/s00415-023-11956-z)
Supplement: Supplementary file 2 — Supplementary file2 (DOCX 61 KB) [file 415_2023_11956_MOESM2_ESM.docx]

| Question | Overall  sample | **Country** | | | | **Expertise** | | | **Setting** | | | |
| --- | --- | --- | --- | --- | --- | --- | --- | --- | --- | --- | --- | --- |
|  |  | Europe | USA | APA | *p* | <10 years | >10 years | *p* | ICU | ISRU | SCF | *p* |
| **1. What DoC diagnostic tools do you use in your practice?** | ***n*=196** | ***n*=80** | ***n*=61** | ***n*=55** |  | ***n*=70** | ***n*=126** |  | ***n*=43** | ***n*=98** | ***n*=28** |  |
| Bedside behavioural examination with standardized tools | 79% | 89% | 79% | 64% | **.002** | 74% | 81% | .276 | 81% | 84% | 89% | .668 |
| Bedside behavioural examination without standardized tools | 34% | 30% | 43% | 31% | .245 | 26% | 33% | .736 | 35% | 36% | 36% | .995 |
| Functional neuroimaging | 19% | 20% | 7% | 33% | **.002** | 20% | 19% | .872 | 21% | 18% | 25% | .734 |
| Structural neuroimaging | 52% | 56% | 43% | 55% | .240 | 49% | 53% | .537 | 56% | 57% | 54% | .943 |
| Neurophysiological evaluation | 54% | 66% | 38% | 53% | **.001** | 41% | 60% | **.011** | 63% | 51% | 57% | .420 |
| Other | 11% | 6% | 11% | 16% | .170 | 10% | 11% | .810 | 14% | 5% | 7% | .191 |
| **2. Do you observe the patient prior to actually doing your hands-on clinical assessment as recommended by CRS-R?** | ***n*=196** | ***n*=80** | ***n*=61** | ***n*=55** |  | ***n*=70** | ***n*=126** |  | ***n*=43** | ***n*=98** | ***n*=28** |  |
| Always | 76% | 86% | 72% | 65% | .056 | 73% | 78% | .753 | 72% | 86% | 75% | .395 |
| Sometimes | 15% | 7% | 21% | 22% |  | 16% | 15% |  | 19% | 10% | 18% |  |
| Never | 4% | 2% | 2% | 8% |  | 4% | 3% |  | 4% | 2% | 7% |  |
| None of the above | 5% | 5% | 5% | 5% |  | 7% | 4% |  | 5% | 2% | 0% |  |
| **3. Do you usually try to prime the patient’s arousal level before assessing their level of consciousness?** | ***n*=166** | ***n*=67** | ***n*=53** | ***n*=46** |  | ***n*=54** | ***n*=112** |  | ***n*=40** | ***n*=83** | ***n*=25** |  |
| Yes | 72% | 73% | 62% | 83% | .077 | 78% | 70% | .273 | 77% | 73% | 68% | .698 |
| No | 28% | 27% | 38% | 17% |  | 22% | 30% |  | 23% | 27% | 32% |  |
| **3.1. If yes, how?** | ***n*=114** | ***n*=49** | ***n*=31** | ***n*=34** |  | ***n*=39** | ***n*=75** |  | ***n*=30** | ***n*=61** | ***n*=15** |  |
| Auditory stimulation | 83% | 84% | 74% | 91% | .185 | 82% | 84% | .791 | 80% | 87% | 73% | .399 |
| Tactile stimulation | 79% | 77% | 81% | 79% | .944 | 74% | 81% | .386 | 77% | 79% | 73% | .901 |
| Noxious stimulation | 48% | 49% | 45% | 50% | .918 | 41% | 52% | .266 | 40% | 52% | 33% | .298 |
| Arousal facilitation protocol | 40% | 33% | 52% | 41% | .241 | 44% | 39% | .611 | 37% | 46% | 27% | .348 |
| Manual eye opening | 39% | 35% | 39% | 44% | .686 | 41% | 37% | .701 | 50% | 36% | 20% | .135 |
| Visual fixation | 26% | 33% | 13% | 29% | .131 | 20% | 29% | .310 | 23% | 28% | 20% | .781 |
| Visual pursuit | 28% | 31% | 26% | 26% | .870 | 28% | 28% | .982 | 33% | 29% | 13% | .354 |
| Other | 11% | 10% | 16% | 9% | .613 | 13% | 11% | .731 | 17% | 8% | 13% | .471 |
| **4. Is it possible in your centre to integrate expert clinical evaluation, EEG-based techniques, and/or functional neuroimaging for the evaluation of patients with DoCs?** | ***n*=158** | ***n*=66** | ***n*=51** | ***n*=41** |  | ***n*=50** | ***n*=108** |  | ***n*=39** | ***n*=81** | ***n*=23** |  |
| Yes | 65% | 62% | 59% | 76% | .213 | 62% | 66% | .648 | 80% | 59% | 52% | **.045** |
| No | 35% | 38% | 41% | 24% |  | 38% | 34% |  | 20% | 41% | 48% |  |

**Supplementary Table 1.** Results of the survey with responses for each question as a function of the country, expertise, and setting.

**Supplementary Table 1.** Results of the survey with percentage of responses for each question as a function of the country, expertise, and setting. *(continued)*

| Question | Overall  sample | **Country** | | | | **Expertise** | | | **Setting** | | | |
| --- | --- | --- | --- | --- | --- | --- | --- | --- | --- | --- | --- | --- |
|  |  | Europe | USA | APA | *p* | <10 years | >10 years | *p* | ICU | ISRU | SCF | *p* |
| **5. Which of the following clinical tools do you usually use?** | ***n*=130** | ***n*=57** | ***n*=40** | ***n*=33** |  | ***n*=40** | ***n*=90** |  | ***n*=32** | ***n*=69** | ***n*=21** |  |
| CRS-R | 75% | 82% | 82% | 54% | **.006** | 85% | 71% | .090 | 69% | 87% | 57% | **.008** |
| FOUR | 6% | 5% | 0% | 15% | **.026** | 5% | 6.7% | .715 | 12% | 6% | 0% | .184 |
| NCS-R | 21% | 28% | 15% | 18% | .263 | 25% | 20% | .522 | 9% | 32% | 9% | **.012** |
| GCS | 63% | 63% | 57% | 73% | .399 | 52% | 69% | .073 | 75% | 62% | 52% | .224 |
| SECONDs | 11% | 3% | 12% | 21% | **.030** | 22% | 6% | **.004** | 6% | 10% | 19% | .329 |
| Other | 22% | 28% | 20% | 15% | .335 | 15% | 26% | .182 | 12% | 27% | 19% | .222 |
| **6. Which of the following technological assessments of consciousness do you use in your clinical or research practice?** | ***n*=131** | ***n*=57** | ***n*=41** | ***n*=33** |  | ***n*=41** | ***n*=90** |  | ***n*=32** | ***n*=69** | ***n*=21** |  |
| H20-PET | 4% | 0% | 2% | 12% | **.013** | 12% | 0% | **.001** | 3% | 3% | 5% | .914 |
| FDG-PET | 15% | 17% | 10% | 18% | .495 | 15% | 16% | .892 | 12% | 13% | 24% | .436 |
| Resting state fMRI | 29% | 30% | 17% | 42% | .057 | 29% | 29% | .965 | 28% | 33% | 14% | .239 |
| Active fMRI | 16% | 16% | 7% | 27% | .067 | 22% | 13% | .213 | 6% | 19% | 14% | .252 |
| Standard EEG (Quantitative analysis) | 54% | 58% | 49% | 54% | .670 | 46% | 58% | .223 | 62% | 52% | 43% | .360 |
| Standard EEG (Qualitative analysis) | 47% | 58% | 37% | 39% | .072 | 44% | 48% | .680 | 53% | 51% | 33% | .310 |
| Sleep EEG (Quantitative analysis) | 14% | 12% | 17% | 12% | .756 | 2% | 19% | **.011** | 25% | 9% | 9% | .068 |
| Sleep EEG (Qualitative analysis) | 15% | 19% | 12% | 12% | .531 | 10% | 18% | .237 | 28% | 12% | 9% | .072 |
| High-Density EEG (Quantitative analysis) | 8% | 12% | 2% | 6% | .180 | 12% | 6% | .184 | 6% | 7% | 9% | .904 |
| High-Density EEG (Qualitative analysis) | 6% | 9% | 0% | 9% | .143 | 7% | 6% | .696 | 6% | 9% | 0% | .369 |
| Somatosensory evoked potentials | 43% | 58% | 27% | 36% | **.006** | 32% | 48% | .085 | 47% | 48% | 29% | .281 |
| Brain-stem evoked potentials | 38% | 49% | 22% | 39% | **.024** | 24% | 44% | **.028** | 41% | 41% | 33% | .825 |
| Event-related potentials | 18% | 21% | 7% | 27% | .068 | 24% | 16% | .225 | 12% | 20% | 19% | .633 |
| Transcranial Magnetic Stimulation (TMS)-EEG | 15% | 12% | 12% | 21% | .449 | 22% | 11% | .102 | 16% | 13% | 19% | .784 |
| Brain Computer Interface | 2% | 2% | 0% | 3% | .562 | 2% | 1% | .565 | 0% | 3% | 0% | .458 |
| Other | 22% | 16% | 37% | 15% | **.027** | 24% | 21% | .675 | 31% | 20% | 19% | .427 |

**Supplementary Table 1.** Results of the survey with percentage of responses for each question as a function of the country, expertise, and setting. *(continued)*

| Question | Overall  sample | **Country** | | | | **Expertise** | | | **Setting** | | | |
| --- | --- | --- | --- | --- | --- | --- | --- | --- | --- | --- | --- | --- |
|  |  | Europe | USA | APA | *p* | <10 years | >10 years | *p* | ICU | ISRU | SCF | *p* |
| **7. If you use neuroimaging, what kind of paradigm do you perform?** | ***n*=131** | ***n*=57** | ***n*=41** | ***n*=33** |  | ***n*=41** | ***n*=90** |  | ***n*=32** | ***n*=69** | ***n*=21** |  |
| Resting state | 56% | 56% | 39% | 79% | **.003** | 58% | 56% | .750 | 50% | 62% | 43% | .218 |
| Passive sensory stimulation | 14% | 9% | 5% | 33% | **.001** | 24% | 9% | **.017** | 12% | 17% | 5% | .330 |
| Active tasks | 18% | 18% | 10% | 30% | .074 | 27% | 14% | .089 | 12% | 25% | 9% | .170 |
| None of the above | 37% | 37% | 54% | 18% | **.007** | 32% | 40% | .363 | 47% | 29% | 52% | .070 |
| **8. How often is the clinical assessment repeated in your diagnostic protocol?** | ***n*=131** | ***n*=57** | ***n*=41** | ***n*=33** |  | ***n*=41** | ***n*=90** |  | ***n*=32** | ***n*=69** | ***n*=21** |  |
| Regularly and equally for all the patients | 35% | 39% | 24% | 42% | .207 | 41% | 32% | .304 | 31% | 42% | 24% | .252 |
| It depends on the patient’s condition | 63% | 56% | 63% | 73% | .290 | 58% | 64% | .517 | 69% | 56% | 76% | .195 |
| It depends on the available qualified staff | 17% | 12% | 22% | 18% | .437 | 17% | 17% | .954 | 9% | 17% | 29% | .194 |
| At admission only | 5% | 0% | 15% | 3% | **.005** | 12% | 2% | **.019** | 6% | 4% | 5% | .918 |
| At admission and discharge | 8% | 9% | 5% | 9% | .724 | 7% | 8% | .927 | 9% | 7% | 0% | .379 |
| **9. Do you practice regular patient follow-ups?** | ***n*=131** | ***n*=57** | ***n*=41** | ***n*=33** |  | ***n*=41** | ***n*=90** |  | ***n*=32** | ***n*=69** | ***n*=21** |  |
| Yes | 60% | 44% | 71% | 76% | **.018** | 63% | 59% | .708 | 78% | 54% | 52% | .056 |
| No | 8% | 10% | 7% | 3% |  | 5% | 9% |  | 3% | 10% | 0% |  |
| Not always | 32% | 46% | 22% | 21% |  | 32% | 32% |  | 19% | 36% | 48% |  |
| **10. What are the factors impacting the frequency of the follow-ups?** | ***n*=129** | ***n*=57** | ***n*=40** | ***n*=32** |  | ***n*=41** | ***n*=88** |  | ***n*=31** | ***n*=68** | ***n*=21** |  |
| Caregivers’ availability/willingness/involvement | 55% | 51% | 52% | 66% | .377 | 51% | 57% | .552 | 45% | 57% | 62% | .413 |
| Physical (i.e., distance) feasibility | 64% | 61% | 55% | 78% | .116 | 51% | 69% | **.047** | 55% | 72% | 52% | .117 |
| Family-medical team relationship | 26% | 19% | 20% | 47% | **.010** | 27% | 26% | .934 | 32% | 28% | 9% | .154 |
| Other | 37% | 40% | 40% | 28% | .471 | 41% | 35% | .495 | 39% | 37% | 43% | .881 |
| **11. Has the COVID-19 pandemic changed your practice in this regard?** | ***n*=129** | ***n*=57** | ***n*=40** | ***n*=32** |  | ***n*=41** | ***n*=88** |  | ***n*=31** | ***n*=68** | ***n*=21** |  |
| Yes | 55% | 58% | 40% | 69% | **.043** | 51% | 57% | .552 | 48% | 62% | 48% | .326 |
| No | 45% | 42% | 60% | 31% |  | 49% | 43% |  | 52% | 38% | 52% |  |

| Question | Overall  sample | **Country** | | | | **Expertise** | | | **Setting** | | | |
| --- | --- | --- | --- | --- | --- | --- | --- | --- | --- | --- | --- | --- |
|  |  | Europe | USA | APA | *p* | <10 years | >10 years | *p* | ICU | ISRU | SCF | *p* |
| **12. What is the main challenge in the implementation of a multimodal assessment of consciousness in your program with regards to utilization of high-density EEG, PET and fMRI?** | ***n*=113** | ***n*=44-48** | ***n*=22-32** | ***n*=21-25** |  | ***n*=30-35** | ***n*=56-67** |  | ***n*=18-23** | ***n*=50-56** | ***n*=10-15** |  |
| Collaboration by the patient | 3.6±2.0 | 3.9±2.3 | 4.6±1.9 | 4.2±2.1 | .380 | 5.0±2.1 | 3.7±2.1 | **.009** | 4.1±1.8 | 4.4±2.3 | 3.4±2.1 | .309 |
| Logistics | 3.8±1.7 | 3.7±1.8 | 4.1±1.4 | 3.5±1.7 | .366 | 3.2±1.6 | 4.0±1.7 | **.029** | 4.1±1.6 | 3.6±1.7 | 3.9±1.7 | .484 |
| Neurosurgical issues | 4.2±1.9 | 4.0±1.8 | 4.2±2.1 | 4.6±2.0 | .520 | 4.8±2.0 | 3.9±1.9 | **.043** | 4.4±1.9 | 4.0±1.8 | 4.6±2.5 | .579 |
| Cost | 3.6±2.0 | 4.0±2.0 | 3.4±2.0 | 3.2±2.1 | .304 | 3.5±2.0 | 3.7±2.1 | .607 | 2.8±1.9 | 4.1±2.1 | 2.9±1.6 | **.028** |
| Medical stability of the patient | 3.7±1.8 | 3.7±1.6 | 3.8±1.9 | 3.7±1.9 | .928 | 4.6±1.5 | 3.3±1.7 | **.001** | 3.8±2.2 | 3.9±1.5 | 3.4±1.9 | .657 |
| Availability of the technique | 3.6±2.2 | 4.1±2.2 | 3.1±2.3 | 3.5±1.9 | .173 | 3.0±2.0 | 4.0±2.2 | **.032** | 3.8±2.3 | 3.6±2.3 | 3.5±2.2 | .920 |
| Level of necessary skills and expertise | 3.4±1.8 | 3.5±1.8 | 3.0±1.8 | 3.5±1.8 | .444 | 3.3±1.8 | 3.4±1.8 | .859 | 3.1±1.8 | 3.4±1.9 | 3.5±1.4 | .726 |
| **13. Do you or your team regularly counsel families or patient’s representatives/caregivers about the patient’s diagnosis, prognosis and possible long-term care options?** | ***n*=107** | ***n*=48** | ***n*=33** | ***n*=26** |  | ***n*=34** | ***n*=73** |  | ***n*=25** | ***n*=57** | ***n*=16** |  |
| Always | 86% | 90% | 82% | 86% | .581 | 79% | 89% | .203 | 72% | 96% | 75% | **.012** |
| Sometimes | 13% | 10% | 15% | 14% |  | 18% | 11% |  | 24% | 4% | 25% |  |
| Never | 1% | 0% | 3% | 0% |  | 3% | 0% |  | 4% | 0% | 0% |  |
| **13.1. If yes, when do you provide said counseling?** | ***n*=93** | ***n*=43** | ***n*=28** | ***n*=22** |  | ***n*=28** | ***n*=65** |  | ***n*=19** | ***n*=55** | ***n*=12** |  |
| At admission | 57% | 63% | 43% | 64% | .195 | 57% | 57% | .984 | 63% | 54% | 67% | .655 |
| At discharge | 58% | 63% | 46% | 64% | .328 | 46% | 63% | .136 | 63% | 60% | 42% | .446 |
| After discharge | 30% | 30% | 25% | 36% | .685 | 36% | 28% | .439 | 32% | 29% | 25% | .926 |
| During their stay | 92% | 95% | 93% | 86% | .428 | 86% | 95% | .105 | 95% | 96% | 83% | .216 |
| **14. Has the COVID-19 pandemic changed your practice in this regard?** | ***n*=93** | ***n*=43** | ***n*=28** | ***n*=22** |  | ***n*=28** | ***n*=65** |  | ***n*=19** | ***n*=55** | ***n*=12** |  |
| Yes | 45% | 67% | 25% | 27% | **<.001** | 36% | 49% | .230 | 32% | 49% | 50% | .393 |
| No | 55% | 33% | 75% | 73% |  | 64% | 51% |  | 68% | 51% | 50% |  |

**Supplementary Table 1.** Results of the survey with percentage of responses for each question as a function of the country, expertise, and setting. *(continued)*

**Supplementary Table 1.** Results of the survey with percentage of responses for each question as a function of the country, expertise, and setting. *(continued)*

| Question | Overall  sample | **Country** | | | | **Expertise** | | | **Setting** | | | |
| --- | --- | --- | --- | --- | --- | --- | --- | --- | --- | --- | --- | --- |
|  |  | Europe | USA | APA | *p* | <10 years | >10 years | *p* | ICU | ISRU | SCF | *p* |
| **15. Do you or your team provide evidence based prognosis information to family members of patients with DoC (e.g. as related to type of acquired brain injury, time post onset, neuropathology, etc.)?** | ***n*=109** | ***n*=48** | ***n*=35** | ***n*=26** |  | ***n*=34** | ***n*=75** |  | ***n*=25** | ***n*=57** | ***n*=18** |  |
| Yes | 92% | 85% | 97% | 96% | .103 | 94% | 91% | .544 | 92% | 96% | 78% | **.039** |
| No | 8% | 15% | 3% | 4% |  | 6% | 9% |  | 8% | 4% | 22% |  |
| **16. Do you or your team involve patients’ families in the clinical assessment?** | ***n*=109** | ***n*=48** | ***n*=35** | ***n*=26** |  | ***n*=34** | ***n*=75** |  | ***n*=25** | ***n*=57** | ***n*=18** |  |
| Yes | 71% | 62% | 86% | 65% | .057 | 65% | 73% | .360 | 72% | 74% | 72% | .984 |
| No | 29% | 38% | 14% | 35% |  | 35% | 27% |  | 28% | 26% | 28% |  |
| **17. Do you or your team involve patients’ families in the rehabilitation program?** | ***n*=109** | ***n*=48** | ***n*=35** | ***n*=26** |  | ***n*=34** | ***n*=75** |  | ***n*=25** | ***n*=57** | ***n*=18** |  |
| Always | 63% | 60% | 74% | 54% | .502 | 50% | 69% | .083 | 56% | 72% | 44% | .073 |
| Sometimes | 32% | 35% | 23% | 38% |  | 41% | 28% |  | 44% | 25% | 50% |  |
| Never | 4% | 2% | 3% | 8% |  | 9% | 1% |  | 0% | 3% | 0% |  |
| None of the above | 1% | 3% | 0% | 0% |  | 0% | 2% |  | 0% | 0% | 6% |  |
| **18. Has the COVID-19 pandemic changed your practice in this regard?** | ***n*=109** | ***n*=48** | ***n*=35** | ***n*=26** |  | ***n*=34** | ***n*=75** |  | ***n*=25** | ***n*=57** | ***n*=18** |  |
| Yes | 61% | 71% | 54% | 50% | .141 | 65% | 59% | .550 | 48% | 70% | 67% | .151 |
| No | 39% | 29% | 46% | 50% |  | 35% | 41% |  | 52% | 30% | 33% |  |
| **19. Do you or your team attempt to identify patient and family treatment preferences (e.g., therapeutic and palliative interventions) soon after admission (i.e., within the first 2 weeks)?** | ***n*=109** | ***n*=48** | ***n*=35** | ***n*=26** |  | ***n*=34** | ***n*=75** |  | ***n*=25** | ***n*=57** | ***n*=18** |  |
| Yes | 77% | 73% | 83% | 77% | .568 | 76% | 77% | .921 | 96% | 70% | 78% | **.034** |
| No | 23% | 27% | 17% | 23% |  | 24% | 23% |  | 4% | 30% | 22% |  |

**Supplementary Table 1.** Results of the survey with percentage of responses for each question as a function of the country, expertise, and setting. *(continued)*

| Question | Overall  sample | **Country** | | | | **Expertise** | | | **Setting** | | | |
| --- | --- | --- | --- | --- | --- | --- | --- | --- | --- | --- | --- | --- |
|  |  | Europe | USA | APA | *p* | <10 years | >10 years | *p* | ICU | ISRU | SCF | *p* |
| **20. Do you or your team inform families about the limitations of existing evidence concerning currently employed (standard) treatment and/or non-validated (e.g., experimental) treatment effectiveness and the related potential risks and harms?** | ***n*=109** | ***n*=48** | ***n*=35** | ***n*=26** |  | ***n*=34** | ***n*=75** |  | ***n*=25** | ***n*=57** | ***n*=18** |  |
| Yes | 89% | 90% | 86% | 92% | .707 | 91% | 88% | .624 | 92% | 90% | 83% | .659 |
| No | 11% | 10% | 14% | 8% |  | 9% | 12% |  | 8% | 10% | 17% |  |
| **21. What methods do you use for assisting with outcome prognostication?** | ***n*=108** | ***n*=47** | ***n*=35** | ***n*=26** |  | ***n*=34** | ***n*=74** |  | ***n*=25** | ***n*=57** | ***n*=17** |  |
| Structural MRI | 68% | 68% | 69% | 65% | .962 | 68% | 67% | .993 | 84% | 65% | 41% | **.016** |
| SPECT | 4% | 2% | 6% | 4% | .696 | 0% | 5% | .167 | 0% | 0% | 6% | .087 |
| Behavioral assessment results | 69% | 74% | 69% | 61% | .512 | 76% | 66% | .283 | 64% | 75% | 65% | .483 |
| Etiology of brain injury | 81% | 83% | 89% | 65% | .066 | 79% | 81% | .839 | 80% | 89% | 53% | **.004** |
| Duration of DoC | 74% | 83% | 80% | 50% | **.005** | 62% | 80% | **.048** | 64% | 86% | 59% | **.021** |
| PET | 10% | 13% | 6% | 11% | .560 | 12% | 9% | .713 | 8% | 10% | 12% | .912 |
| Standard EEG | 58% | 62% | 57% | 54% | .796 | 56% | 59% | .726 | 72% | 61% | 41% | .131 |
| High-density EEG | 6% | 8% | 3% | 4% | .493 | 9% | 4% | .315 | 8% | 2% | 18% | **.049** |
| ERP | 17% | 21% | 6% | 23% | .105 | 23% | 13% | .195 | 16% | 21% | 12% | .648 |
| Age | 76% | 74% | 86% | 65% | .176 | 73% | 77% | .693 | 72% | 82% | 65% | .254 |
| Medical history | 78% | 72% | 91% | 69% | .059 | 73% | 80% | .472 | 88% | 81% | 65% | .176 |
| Medical complications | 80% | 79% | 91% | 65% | **.043** | 68% | 85% | **.036** | 84% | 88% | 59% | **.024** |
| Other | 22% | 23% | 20% | 23% | .928 | 32% | 18% | .086 | 24% | 26% | 12% | .457 |
| **22. Do you have a specific DoC rehabilitation program in your centre?** | ***n*=109** | ***n*=48** | ***n*=35** | ***n*=26** |  | ***n*=34** | ***n*=75** |  | ***n*=25** | ***n*=57** | ***n*=18** |  |
| Yes | 46% | 48% | 40% | 50% | .689 | 41% | 48% | .508 | 20% | 58% | 44% | **.007** |
| No | 54% | 52% | 60% | 50% |  | 59% | 52% |  | 80% | 42% | 56% |  |
| **22.1. If yes, is that about:** | ***n*=51** | ***n*=24** | ***n*=14** | ***n*=13** |  | ***n*=15** | ***n*=36** |  | ***n*=5** | ***n*=34** | ***n*=8** |  |
| Consciousness recovery | 12% | 4% | 29% | 8% | .117 | 13% | 11% | .779 | 20% | 6% | 25% | .463 |
| Functional recovery | 4% | 9% | 0% | 0% |  | 7% | 3% |  | 0% | 6% | 0% |  |
| Both | 84% | 87% | 71% | 92% |  | 80% | 86% |  | 80% | 88% | 75% |  |

**Supplementary Table 1.** Results of the survey with percentage of responses for each question as a function of the country, expertise, and setting. *(continued)*

| Question | Overall  sample | **Country** | | | | **Expertise** | | | **Setting** | | | |
| --- | --- | --- | --- | --- | --- | --- | --- | --- | --- | --- | --- | --- |
|  |  | Europe | USA | APA | *p* | <10 years | >10 years | *p* | ICU | ISRU | SCF | *p* |
| **23. Is the rehabilitation program regularly updated on the basis of repeated assessments of consciousness and/or of functional disability?** | ***n*=50** | ***n*=23** | ***n*=14** | ***n*=13** |  | ***n*=14** | ***n*=36** |  | ***n*=5** | ***n*=33** | ***n*=8** |  |
| Yes | 92% | 100% | 93% | 77% | **.049** | 86% | 94% | .307 | 80% | 97% | 87% | .270 |
| No | 8% | 0% | 7% | 23% |  | 14% | 6% |  | 20% | 3% | 13% |  |
| **24. Do you specifically assess pain in patients with DoCs?** | ***n*=109** | ***n*=48** | ***n*=35** | ***n*=26** |  | ***n*=34** | ***n*=75** |  | ***n*=25** | ***n*=57** | ***n*=18** |  |
| Yes | 76% | 79% | 71% | 77% | .712 | 65% | 81% | .059 | 68% | 75% | 89% | .283 |
| No | 24% | 21% | 29% | 23% |  | 35% | 19% |  | 32% | 25% | 11% |  |
| **24.1. If yes, is that about:** | ***n*=83** | ***n*=38** | ***n*=25** | ***n*=20** |  | ***n*=22** | ***n*=61** |  | ***n*=17** | ***n*=43** | ***n*=16** |  |
| NCS-R | 47% | 60% | 24% | 50% | **.017** | 50% | 46% | .741 | 41% | 56% | 37% | .357 |
| Clinical assessment | 59% | 39% | 80% | 70% | **.003** | 59% | 59% | .995 | 71% | 56% | 62% | .564 |
| Other | 28% | 24% | 28% | 35% | .657 | 36% | 25% | .290 | 35% | 28% | 19% | .568 |
| **25. Would you treat pain if you are unsure about the patient’s level of consciousness based on bedside assessment?** | ***n*=108** | ***n*=4** | ***n*=18** | ***n*=23** |  | ***n*=34** | ***n*=74** |  | ***n*=25** | ***n*=56** | ***n*=18** |  |
| Yes | 87% | 96% | 82% | 77% | **.043** | 85% | 88% | .715 | 80% | 91% | 83% | .350 |
| No | 13% | 4% | 18% | 23% |  | 15% | 12% |  | 20% | 9% | 17% |  |
| **26. Should pain be treated (e.g., through use of pain medications) regardless of the level of residual consciousness?** | ***n*=108** | ***n*=48** | ***n*=34** | ***n*=26** |  | ***n*=34** | ***n*=74** |  | ***n*=25** | ***n*=56** | ***n*=18** |  |
| Yes | 81% | 85% | 76% | 77% | .521 | 68% | 86% | **.022** | 84% | 87% | 56% | **.010** |
| No | 19% | 15% | 23% | 23% |  | 32% | 14% |  | 16% | 13% | 44% |  |
| **27. Do you counsel families about the difficulty of detecting pain in patients with DOCs?** | ***n*=108** | ***n*=48** | ***n*=34** | ***n*=26** |  | ***n*=34** | ***n*=74** |  | ***n*=25** | ***n*=56** | ***n*=18** |  |
| Yes | 81% | 81% | 79% | 81% | .978 | 73% | 84% | .211 | 72% | 86% | 78% | .329 |
| No | 19% | 19% | 21% | 19% |  | 27% | 16% |  | 28% | 14% | 22% |  |
| **28. Do you work collaboratively with other DoC centers/experts?** | ***n*=108** | ***n*=48** | ***n*=34** | ***n*=26** |  | ***n*=34** | ***n*=74** |  | ***n*=25** | ***n*=56** | ***n*=18** |  |
| Yes | 72% | 75% | 62% | 81% | .225 | 82% | 68% | .111 | 68% | 77% | 67% | .583 |
| No | 28% | 25% | 38% | 19% |  | 18% | 32% |  | 32% | 23% | 33% |  |

**Supplementary Table 1.** Results of the survey with percentage of responses for each question as a function of the country, expertise, and setting. *(continued)*

| Question | Overall  sample | **Country** | | | | **Expertise** | | | **Setting** | | | |
| --- | --- | --- | --- | --- | --- | --- | --- | --- | --- | --- | --- | --- |
|  |  | Europe | USA | APA | *p* | <10 years | >10 years | *p* | ICU | ISRU | SCF | *p* |
| **28.1. If yes, in what context?** | ***n*=78** | ***n*=36** | ***n*=21** | ***n*=21** |  | ***n*=28** | ***n*=50** |  | ***n*=17** | ***n*=43** | ***n*=12** |  |
| Clinical | 32% | 28% | 43% | 29% | .704 | 21% | 38% | .209 | 53% | 26% | 33% | .336 |
| Research | 17% | 17% | 19% | 14% |  | 14% | 18% |  | 6% | 18% | 17% |  |
| Both | 51% | 55% | 38% | 57% |  | 65% | 44% |  | 41% | 56% | 50% |  |
| **29. Should the term permanent Vegetative State (VS) or Unresponsive Wakefulness Syndrome (UWS) be replaced with the "VS/UWS and its specific duration"?** | ***n*=106** | ***n*=48** | ***n*=34** | ***n*=24** |  | ***n*=33** | ***n*=73** |  | ***n*=25** | ***n*=55** | ***n*=17** |  |
| Yes | 73% | 71% | 71% | 79% | .717 | 76% | 71% | .628 | 64% | 74% | 82% | .396 |
| No | 27% | 29% | 29% | 21% |  | 24% | 29% |  | 36% | 26% | 18% |  |

Questions 1, 3.1, 5-8, 10, 12, 13.1, 21, 24.1 had multiple-choice answer. Data are reported as percentages and univariate statistics are based upon the χ^2^ test, except for Question 12 for which means±SD are reported and statistics are based upon the Student’s t tests. Significant differences across the groups are reported in bold.

Abbreviations: APA=Asia-Pacific and Africa; ; ICU=Intensive Care Unit; ISRU=Intensive Specialized Rehabilitation Unit; SCF=Specialized Care Facilities; USA=United States of America.
